# Evaluation of an Antimicrobial L-Amino Acid Oxidase and Peptide Derivatives from *Bothropoides mattogrosensis* Pitviper Venom

**DOI:** 10.1371/journal.pone.0033639

**Published:** 2012-03-16

**Authors:** Brunna M. Okubo, Osmar N. Silva, Ludovico Migliolo, Diego G. Gomes, William F. Porto, Carla L. Batista, Carmel S. Ramos, Hortência H. S. Holanda, Simoni C. Dias, Octavio L. Franco, Susana E. Moreno

**Affiliations:** 1 Programa de Pós-Graduação em Biotecnologia, Universidade Católica Dom Bosco, Campo Grande, Mato Grosso do Sul, Brazil; 2 Centro de Análises Proteômicas e Bioquímicas, Pós-Graduação em Ciências Genômicas e Biotecnologia, Brasília, Distrito Federal, Brazil; 3 Programa de Pós-Graduação em Ciências Biológicas (Biotecnologia e Genética), Universidade Federal de Juiz de Fora, Juiz de Fora, Minas Gerais, Brazil; Instituto Butantan, Brazil

## Abstract

Healthcare-associated infections (HAIs) are causes of mortality and morbidity worldwide. The prevalence of bacterial resistance to common antibiotics has increased in recent years, highlighting the need to develop novel alternatives for controlling these pathogens. Pitviper venoms are composed of a multifaceted mixture of peptides, proteins and inorganic components. L-amino oxidase (LAO) is a multifunctional enzyme that is able to develop different activities including antibacterial activity. In this study a novel LAO from *Bothrops mattogrosensis* (*Bm*LAO) was isolated and biochemically characterized. Partial enzyme sequence showed full identity to *Bothrops pauloensis* LAO. Moreover, LAO here isolated showed remarkable antibacterial activity against Gram-positive and -negative bacteria, clearly suggesting a secondary protective function. Otherwise, no cytotoxic activities against macrophages and erythrocytes were observed. Finally, some LAO fragments (*Bm*LAO-f1, *Bm*LAO-f2 and *Bm*LAO-f3) were synthesized and further evaluated, also showing enhanced antimicrobial activity. Peptide fragments, which are the key residues involved in antimicrobial activity, were also structurally studied by using theoretical models. The fragments reported here may be promising candidates in the rational design of new antibiotics that could be used to control resistant microorganisms.

## Introduction

Healthcare-associated infections (HAIs) are causes of mortality and morbidity worldwide. It is estimated that the incidence of HAIs is around 5–10% in developed countries, with a frequency exceeding 25% in developing nations. HAIs can be caused by a number of micro-organisms such as bacteria, fungi and viruses [1]. Nevertheless, the pathogens involved in infectious processes consist mainly of constitutive bacteria from normal human microbiota. These bacteria are innocuous in healthy individuals but are able to cause severe and dangerous infections, mainly in immunocompromised individuals [2]. Moreover, the prevalence of bacterial resistance to common antibiotics has increased in recent years, emphasising the need to develop novel alternatives for controlling these pathogens [2].

Indeed, the pursuit of unusual substances from natural sources has been extensive. Animal venoms are considered important sources of novel therapeutic agents due to their unusual and complex protein composition. Pitviper venoms are composed of a multifaceted mixture of peptides, proteins and inorganic components [3], [4]. Among venomous proteins are included phospholipase A_2_, desintegrins, metalo- and serine proteinases, nerve growth factors (NGFs), L-amino acid oxidases (LAO) and several others [3]. LAO is a classical flavoprotein that catalyzes the oxidative deamination of L-amino acids, converting them into keto acids, ammonia and hydrogen peroxide (H_2_O_2_) [5]. Furthermore, LAO is a multifunctional enzyme that is able to carry out different activities such as decreasing platelet aggregation [6], stimulating edema formation [7], controlling tumorous cells [8] or acting as deleterious factors toward multiple pathogens such as bacteria, protozoa and viruses [3], [9]–[11]. Moreover, multifunctional LAOs could act as host defence for other organisms, since they normally occur in a wide variety of animal fluids such as milk [12] and epidermal mucus [10]. Knowledge of the defensive potential of L-amino-oxidases has recently improved significantly, including the possible mechanism of action. Yang et al. [13], working with a fungal LAO, demonstrated that the enhancing of H_2_O_2_ related to enzyme activity could lead to cell damage, and also lipid peroxidation and DNA strand breakage, causing reduction in bacterial development. Similar antibacterial activity was obtained with LAO isolated from rabbitfish (*Siganus oramin*), suggesting that this enzyme may contribute significantly to the host's non-specific immune defense mechanism to control microorganisms [14]. Moreover, this antimicrobial activity was also reported in LAOs isolated from snake venoms. De Melo Alves et al. [15] showed that LAO isolated from *Bothrops atrox* venom displays remarkable bactericidal activity against both Gram-positive and Gram-negative bacteria in addition to antiprotozoal activity. Similar data were obtained with LAO isolated from venom of *Ophiophagus hannah* [16] in which higher antimicrobial activities with lower MICs were observed.

For this reason, the LAO group has been focused as a possible source of antimicrobial tools for biotechnological and pharmaceutical purposes. In summary, this study presents a novel LAO, isolated from *B. mattogrosensis* (*Bm*LAO) venom. In addition to enzymatic activity, the LAO was evaluated, taking into account its antimicrobial properties, which showed clear activity against Gram-positive and -negative bacteria, clearly suggesting a secondary and promiscuous protective function. Otherwise, no cytotoxic activity against macrophages and erythrocytes was observed. In order to make the use of this protein possible for biotechnological purposes, protein was structurally evaluated and further fragmented in order to reduce the size, maintaining the antimicrobial activity. Complete LAO protein was fragmented according to exposed charges and hydrophobic moment, since most antimicrobial peptides with activity against Gram-positive and –negative bacteria are cationic and show at least one hydrophobic face. LAO fragments yielded were synthesized and were functional, and *in silico* structural analyses produced the first report of LAO fragments with antimicrobial activity.

## Materials and Methods

### Venom extraction

Crude *Bothropoides mattogrosensis* venom was collected from 16 adult specimens from the serpentarium at the Universidade Católica Dom Bosco by direct pressure of snake venom glands. This study was approved by the Animal Use Committee (CEUA) at the Institute of Biological Sciences, University of Brasilia. The venom was pooled, lyophilized and stored at −20°C. The total protein was quantified using the Bradford assay [17].

### L-amino acid oxidase isolation

Firstly, 30 mg.mL^−1^ of freeze-dried *B. mattogrosensis* crude venom was re-suspended in 50 mM Tris-HCl buffer pH 7.5 and filtered through 0.22 µm nylon membranes. Extract was applied onto a Sephacryl S-100 gel filtration chromatograph (Pharmacia, Sweden – 25–75 µm, 600 mm×35 mm) previously equilibrated with the same buffer. The crude venom was eluted with 50 mM Tris-HCl buffer pH 7.5 at a flow rate of 1.8 mL.min^−1^. Fractions of 3.0 ml were collected and monitored at 280 nm. The fractions were dialyzed with membrane cut-off of 3.5 kDa (Spectrum Laboratories, USA) and further lyophilized. The active fractions were quantified using the Bradford assay [17] and monitored through bioassays against Gram-positive and -negative bacteria as described below. Reversed-phase high performance liquid chromatography (RP-HPLC) was performed by using a semi-preparative RP-HPLC C18 (Vydac, USA - 5 µm, 250 mm×10 mm). Retained peptides were eluted with a linear gradient of 5 to 95% acetonitrile/TFA 0.1% (v/v). Chromatography was run for 60 min at a flow rate of 2.5 mL.min^−1^. The experiment was monitored at 280 nm. Fractions were manually collected and lyophilized.

### Mass spectrometry and *de novo* sequencing

Determination of molecular mass peptides was performed by using an UltraFlex II MALDI-ToF/ToF Mass Spectrometer (Bruker Daltonics, USA). The fractions with activity were dissolved in α-cyano-4-hydroxycinnamic acid matrix solution (1∶3, v/v), spotted onto a MALDI target massive plate and dried at room temperature for 15 min. The peptides' monoisotopic mass were obtained in reflector mode with external calibration, using the Peptide Calibration Standard for Mass Spectrometry calibration mixture (up to 4,000 Da mass range, Bruker Daltonics). MS/MS spectra were obtained by LIFT/CID fragmentation. Primary structures of the peptides were manually interpreted, and isomeric and isobaric residues were assigned based on the high-energy fragmentation ions according to [18]. FlexAnalysis 2.4 (Bruker Daltonics) and PepSeq (MicroMass) software were used to interpret mass spectra.

### Sequence analysis

The search for similar sequences was performed by using Blastp program [19] using non-redundant protein database. ClustalW tool [20] was used to calculate the identity and similarity scores among the sequenced peptides, and the primary structure of LAO sequences with three-dimensional structures was experimentally resolved.

### Measurement of LAO activity

Assessment of LAO activity was conducted in 96-well microplates, with 10 µL of enzyme solution per well, and 90 µL^−1^ of substrate was added to initiate the reaction. The reaction medium consisted of a total volume of 100 µL containing 10 mM Tris-HCl buffer pH 7.4, 250 µM L-leucine, 2 µM o-phenylenediamine (OPD), 0.81 U.mL-1 of hydrogen peroxide (H_2_O_2_). After incubation at 37°C for 20 min, the reaction was finished by adding 50 µL of 2M H_2_SO_4_. The absorbance of the reaction mixture was measured at 490 nm, and enzyme activity was expressed as the increase in absorbance after the incubation time [21].

### Solid-phase peptide synthesis

After LAO sequencing, some internal peptides were selected for chemical synthesis. These peptides, obtained by MALDI ToF/ToF analysis, were selected on the basis of 20 to 30% of theoretical hydrophobicity and charges from 0 to +3 in pH 7.0. Parameters were calculated by using Protein Calculator v3.3 server (http://www.scripps.edu/~cdputnam/protcalc.html). Selected peptides were synthesized by stepwise solid-phase with the N-9-fluorenylmethyloxycarbonyl (Fmoc) strategy [22] using a Rink amide resin (0.4 mmol.g^−1^). Side chain protecting groups (t-butyl for threonine and (triphenyl)methyl for histidine) were added. Couplings were performed with 1,3-diisopropylcarbodiimide/1-hydroxybenzotriazole in N,N-dimethylformamide (DMF) for 90 min. Deprotections (15 min, twice) were conducted by piperidine∶DMF solution (1∶4; v∶v). Cleavage from the resin and final deprotection were performed with TFA/water/1,2-ethanedithiol (EDT)/triisopropylsilane (TIS), 94.0/2.5/2.5/1.0, by volume) at room temperature for 90 min. After precipitating the product of cleavage with cold diisopropyl ether the crude peptide was extracted with distilled water and acetonitrile at 50% by volume. The extracted peptide was freeze-dried and further lyophilized for purification. The synthetic peptide concentrations for all experiments *in vitro* were measured as described by Murphy and Kies [23] using ABS_215_ and ABS_225_ nm. No peptide presented tryptophan in its sequences.

### Determination of minimal inhibitory concentration (MIC)

The minimal inhibitory concentration (MIC) was determined using a standardized dilution method according to NCSLA guidelines [24]. Overnight colonies of *Bacillus subtilis* (ATCC 6633), *Enterococcus faecalis* (ATCC 12953), *Staphylococcus aureus* (ATCC29213), *Streptococcus pyogenes* (ATCC19615), *Escherichia coli* (ATCC8739), *Klebsiella pneumonia* (ATCC13885), *Proteus mirabilis* (ATCC25933), *Pseudomonas aeruginosa* ATCC 15442 and *Salmonella typhimurium* (ATCC14028) were suspended to a turbidity of 0.5 units and further diluted in Mueller-Hinton broth (MH) (Himedia - India). For determination of MIC, protein was used in graded concentrations (0, 1, 2, 4, 8, 16, 32, 64, 128, 256 and 512 µg.mL^−1^) from a stock solution. Ten microliters of each concentration was added to each corresponding well of a 96-well plate (TPP, Switzerland) and 1×10^5^ bacteria in the volume of 90 µL. The plate was incubated at 37°C for 12 h.

### Hemolysis assay

Human heparine-blood was obtained from the Universidade Católica de Brasilia cell collection. Collection was obtained with written informed consent. Heparine blood cells were centrifuged at 800 g for 10 min. The erythrocytes were washed three times and re-suspended in 0.1% PBS, pH 7.4. The 10% erythrocyte suspension (v/v) was further incubated for 1 h at 37°C in the presence of fractions F-44, F-51, F-68, *Bm*LAO (0–512 µg.mL^−1^) and fragments *Bm*LAO-f1, *Bm*LAO-f2 and *Bm*LAO-f3 at different concentrations (0–340 µM). The positive control was 0.1% triton X-100 (Sigma-Aldrich, USA). The samples were then centrifuged at 800 g for 10 min. The optical density at 540 nm of the supernatant was measured. The relative optical density compared to that of the suspension treated with 0.1% triton X-100 defined hemolysis percent [25].

### Cell cytotoxicity

Sterile filtered MTT (3-(4,5-dimethylthiazolyl)-2,5-diphenyl-tetrazoliumbromide; Sigma-Aldrich, USA) solution (5 mg.mL^−1^ in PBS) was dark stored at −20°C. RAW 264.7 murine macrophage-like cells, 1×10^5^ cells/well was seeded in 96 well plates (TPP, Switzerland), in supplemented DMEM medium (4 mM glutamine, 10% FCS and 100 units.ml^−1^ penicillin/streptomycin) containing multiple treatments. Treatments consisted of fractions F-44, F-51, F-68, *Bm*LAO (0–512 µg.mL^−1^) and fragments *Bm*LAO-f1, *Bm*LAO-f2 and *Bm*LAO-f3 at different concentrations (0–500 µM). After overnight incubation, 60% of medium was then removed, and 10 µl of the MTT solution was added to each well; the plates were then incubated for 4 h in the presence of 5% CO_2_ at 37°C. The blue formazan product generated was dissolved by the addition of 100 µl of 100% DMSO (Mallinckrodt Chemical) per well. The plates were then gently swirled for 5 min at room temperature in order to dissolve the precipitate. The absorbance was monitored at 575 nm in a Biotek Powerwave HT microplate spectrophotometer (Bio-Tek Instruments, USA) [26].

### *In silico* molecular modelling

The LAO from *Vipera ammodytes ammodytes* (PDB 3kve), resolved through X-ray diffraction presenting a crystal with resolution of 2.57 Å [27], was used as template for molecular model construction. The fragments showed 100% of identity with acquired sequences. Two hundred theoretical three-dimensional structures for each peptide were constructed using the previously described Modeller v.9.8 [28] as a template. The final models were evaluated for their geometric, stereochemical, and energy distributions by using PROCHECK [29]. In addition, RMSD was calculated by overlap of Cα traces and backbones onto the template structure through the program 3DSS [30]. The electrostatic surfaces were calculated with the ABPS tool [31]. The protein structures were visualized and analyzed on Swiss PDB viewer v.3.7 [32] and Delano Scientific's PYMOL (http://pymol.sourceforge.net/).

### Statistical analysis

The results are presented as mean ± SD. The statistical significance of the experimental results was determined by Student's t-test. Values of p<0.05 were considered statistically significant. Prism version 5.0 was used for all statistics.

## Results

### Antibacterial activity of *B. mattogrosensis* crude venom

Based on the perspective of find novel antimicrobial agents in snake venoms the antibacterial activity of *B. mattogrosensis* crude venom (CV*Bm*) against bacteria pathogenic to humans was evaluated. Our results demonstrated that *B. mattogrosensis* crude venom was active against Gram-positive and -negative bacteria (Table 1). *B. mattogrosensis* venom showed activity against Gram-negative bacteria, being most active against *K. pneumoniae* with MIC of 64 µg.mL^−1^, followed by deleterious activity obtained against *E. coli* and *S. typhimurium* with MICs of 128 µg.mL^−1^. On the other hand, venom was also evaluated toward *P. mirabilis*, *P. aeruginosa* and all other Gram-positive bacteria, showing lower antimicrobial effectiveness with MICs greater than 256 µg.mL^−1^.

**Table 1:** Antibacterial activity of CVBm, fractions obtained by gel filtration, HPLC chromatography and fragments obtained after *de novo* sequencing.

Microorganisms	MIC* (µg.mL^−1^)							
	CV*Bm*	Fractions			Fragment (µM/µg.mL^−1^)			
		F-44	F-51	F-68	*Bm*LAO	*Bm*LAO-f1	*Bm*LAO-f2	*Bm*LAO-f3
**Gram-positive**								
*Bacillus subtilis* (ATCC 6633)	512	64	32	128	32	43/64	250/256	220/256
*Enterococcus faecalis* (ATCC 12953)	512	64	32	128	32	43/64	125/128	110/128
*Staphylococcus aureus* (ATCC29213)	256	128	32	128	32	43/64	250/256	220/256
*Streptococcus pyogenes* (ATCC19615)	256	64	32	64	8	43/64	125/128	110/128
**Gram-negative**								
*Escherichia coli* (ATCC8739)	128	64	16	64	4	22/32	250/256	220/256
*Klebsiella pneumoniae* (ATCC13885)	64	32	8	64	2	22/32	125/128	110/128
*Proteus mirabilis* (ATCC25933)	256	32	16	38	2	43/64	125/128	110/128
*Pseudomonas aeruginosa* (ATCC15442)	256	32	8	64	8	22/32	250/256	220/256
*Salmonella typhimurium* (ATCC14028)	128	64	16	64	8	43/64	250/128	220/256

* MIC (Minimum Inhibitory Concentration) is defined as the lowest concentration that inhibited 100% of bacterial growth.

Inhibition by each sample was determined after 12 h incubation. **CV** ***Bm***: *B. mattogrosensis* crude venom; ***Bm*** **LAO**: *B. mattogrosensis* L-amino acid oxidase; ***Bm*** **LAO-f1, -f2 and -f3**: *B. mattogrosensis* L-amino acid oxidase fragments 1, 2 and 3.

### Purification of antibacterial protein from crude venom of the snake *B. mattogrosensis*

**Figure 1:**
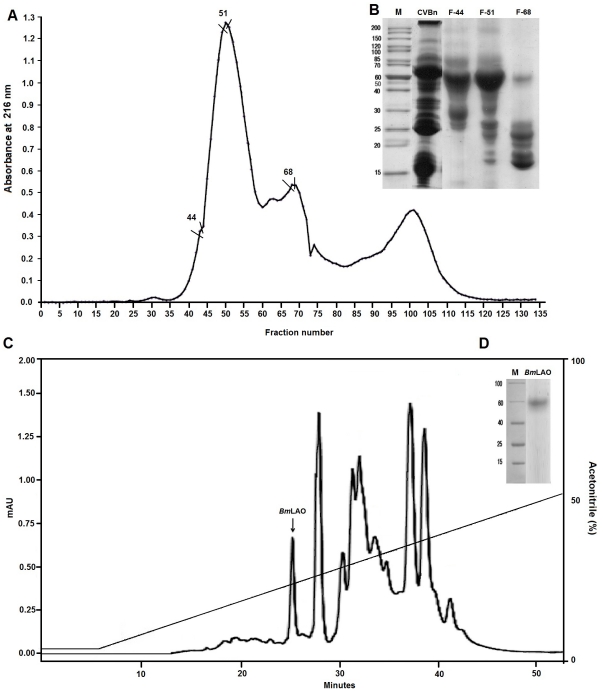
Purification process of antibacterial protein from *Bothrops mattogrosensis* venom. (**A**) Sephacryl S-100 chromatography profile. Thirty milligrams of CVBm (crude venom of *B. mattogrosensis*) was applied on to the column at a flow rate of 1.8 mL.min^−1^. The protein content was monitored by measuring the absorbance at 280 nm. (B) SDS-PAGE of CVBm (C) and gel filtration fractions (44, 51 and 68) with antibacterial activities. M corresponds to molecular mass markers. (C) Chromatographic profile of Fraction (51) applied onto a RP-HPLC C-18. Diagonal line represents a linear acetonitrile gradient (0–100%). Proteins were eluted at a flow rate of 2.5 mL.min^−1^.

Aiming to identify the antibacterial component from CV*Bm*, crude venom was applied onto a gel filtration chromatograph (Figure 1A). Protein elution was monitored by measuring the absorbance at 280 nm, yielding three major protein peaks. Antibacterial activity was further assayed by using all peak fractions (Table 1). The fractions that showed highest antibacterial activity were fractions F-44, F-51 and F-68 (Table 1). These fractions were able to inhibit Gram-positive and -negative bacteria, with fraction F51 being highly effective against pathogens with lower MICs (8 µg.mL^−1^) against *K. pneumoniae* and *P. aeruginosa* (Table 1). These data are similar to previous results found for CV*Bm* (Table 1), which showed higher activity against Gram-negative bacteria. Three fractions from gel filtration chromatography with antibacterial activities (F-44, F-51 and F-68) had their apparent molecular masses evaluated by SDS-PAGE. Fractions F-44, F-51 and F-68 showed a main protein with ∼60 kDa and several contaminants (Figure 1B).

Fraction 51 that showed highest antibacterial activity was further applied into a reversed-phase HPLC chromatograph, generating 17 fractions (Figure 1C). Fraction 7 (*Bm*LAO) showed higher antibacterial activity in comparison to all others evaluated (Table 1). This fraction was capable of efficiently inhibiting the development of *K. pneumoniae* and *P. mirabilis* with MICs of (2 µg.mL^−1^). Moreover, as expected, fraction 7 was more active against Gram-negative bacteria and it was possible to acquire fragments for *de novo* sequencing. The fragments were fragmented by LIFT on MALDI ToF/ToF and *de novo* sequencing (Figure S1). The partial sequence of the enzyme isolated from the venom of *B. mattogrosensis* presented 100% of identity with LAO previously isolated from *B. pauloensis* venom [3].

**Figure 2:**
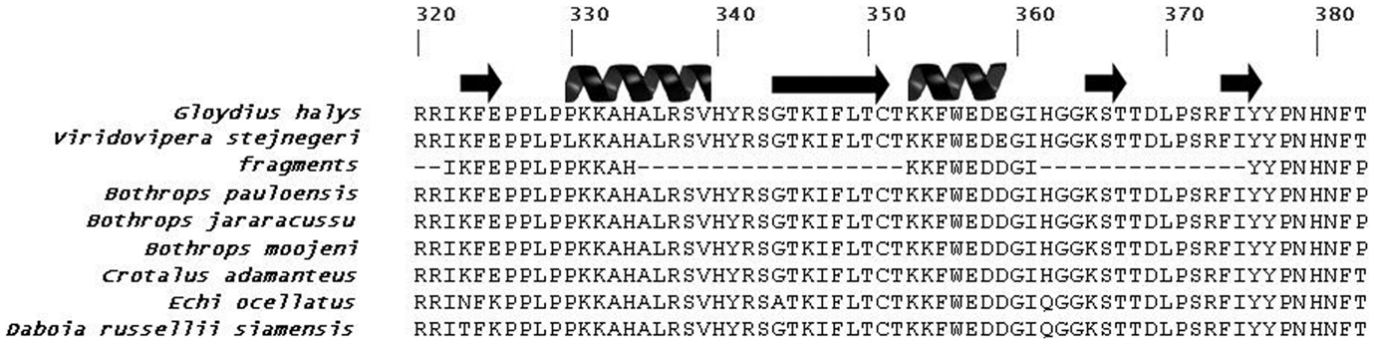
Multiple alignments of protein fragments determined by MALDI ToF/ToF of BmLAO against a wide diversity of L-amino oxidases from different snake species. The black coils represent α-helices and black arrows β-sheets. Numbers represent the positions of amino acid residues obtained from *Gloydius halys* (pdb 1tdk) L-amino-oxidase.

After *de novo* sequencing, the fragments obtained and selected for synthesis were denominated *Bm*LAO-f1 (IKFEPPLPPKKAH-NH_2_), *Bm*LAO-f2 (KKFWEDDG-NH_2_) and *Bm*LAO-f3 (IYYPPNHNFP-NH_2_), respectively, in accordance with alignment (Figure 2). These three peptide fragments were chemically synthesized and further evaluated toward Gram-positive and -negative bacteria. Peptide fragments *Bm*LAO-f1, f2 and f3 showed MIC values from 20 to 45, 125 to 250 and 110 to 220 µM for Gram-positive and –negative bacteria described in this work, and *Bm*LAO-f1 revealed the highest activity (Table 1). In summary, natural protein *Bm*LAO showed higher activity against microorganisms in comparison to synthetic peptides (*Bm*LAO-f1, *Bm*LAO-f2 and *Bm*LAO-f3). Fragments are also interesting as biotechnological products due to the low cost incurred by their small size for synthesis facilities.

### Evaluation of LAO activity

Comparing the molecular mass of protein found in *Bm*LAO with the literature, there is strong evidence that the protein found in this fraction was an L-amino oxidase (LAO). LAOs are able to convert L-amino acids into keto acids, ammonia and H_2_O_2_. LAO activity was determined by monitoring H_2_O_2_ production [33]. Table 2 shows that *Bm*LAO presented clear enzymatic activity in the presence of different amino acid substrates, with the highest activity observed in the presence of L-leucine (340±2.5) followed by His (322±8.2), a classic substrate of LAO [33]. Otherwise, threonines and cysteines were unaffected by the LAO reported here.

**Table 2:** Enzyme activity of *B. mattogrosensis* L-amino acid oxidase (Bm-LAO).

Aminoacid	Specific activity (U.mg^−1^)
Leu	340±2.5*
His	322±8.2**
Phe	244±6.4
Thr	7±3.2
Cys	5±4.0

Values are expressed mean ± S.D. for three independent experiments.

* p<0.05, compared to negative control (PBS buffer, pH 7.4);

** p<0.01 compared to negative control.

### Evaluation of cytotoxicity

The *in vitro* cytotoxicity of F-44, F-51, F-68, *Bm*LAO, *Bm*LAO-f1, *Bm*LAO-f2 and *Bm*LAO-f3 was studied with RAW264.7 and human erythrocytes (Table 3). The *in vitro* cytotoxicity studies confirmed that *Bm*LAO, *Bm*LAO-f1, *Bm*LAO-f2 and *Bm*LAO-f3 showed less than 25% haemolysis and over 78% of cell viability even at higher concentrations.

**Table 3:** Cytotoxic activities of Bm-LAO, fractions 7, 44, 51, 68 the fragments 1, 2 and 3 against RAW264.7 cells and human erythrocytes.

Sample	Cytotoxicity	
	*MTT assay*	*Hemolysis assay*
	*% of control*	
CVBm	20.0±2.7*	18.0±2.5*
*Bm*-LAO	18.1±3.7*	12.5±5.3*
Fraction 7	22.4±2.5*	17.3±4.1*
Fraction 44	13.6±4.5*	6.4±3.8*
Fraction 51	11.2±2.1*	16.5±2.7*
Fraction 68	20.5±3.5*	22.9±2.9*
*Bm*LAO-f1	21.0±3.5*	13.75±3.6*
*Bm*LAO-f2	13.6±4.5*	11.32±2.9*
*Bm*LAO-f3	11.2±2.1*	9.87±3.1*

Samples (512 µg.mL^−1^) were incubated with the cells for 24 h. Cell viability was assessed by MTT assay and hemolytic assay. Values are expressed mean ± S.D. for three independent experiments.

* p<0.05, compared to negative control (PBS buffer, pH 7.4). CVBm: *B. mattogrosensis* crude venom; Bm-LAO: *B. mattogrosensis* L-amino acid oxidase.

### Structural analyses of fragment peptides

**Figure 3:**
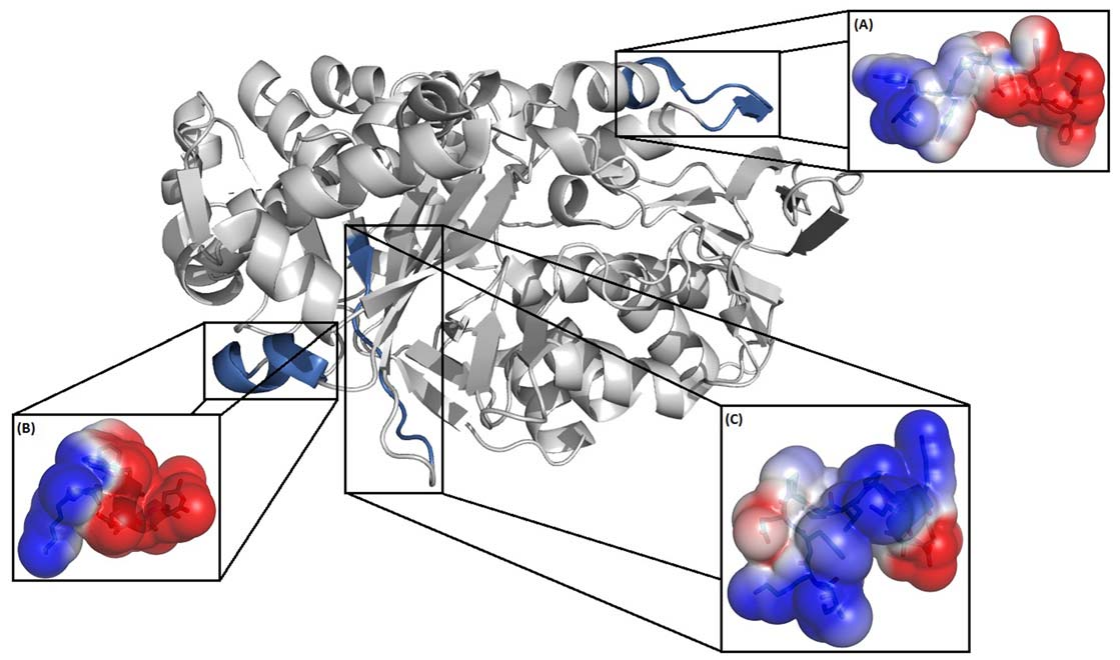
Three-dimensional structure of L-amino-oxidase from *Vipera ammodytes ammodytes* (pdb 3kve) and theoretical models of peptide derivatives (A) BmLAO-f1, (B) BmLAO-f2 and (C) BmLAO-f3. Blue and red regions correspond to the cationic and anionic areas, respectively. The structure was visualized using PyMOL (http://pymol.sourceforge.net/).

**Figure 4:**
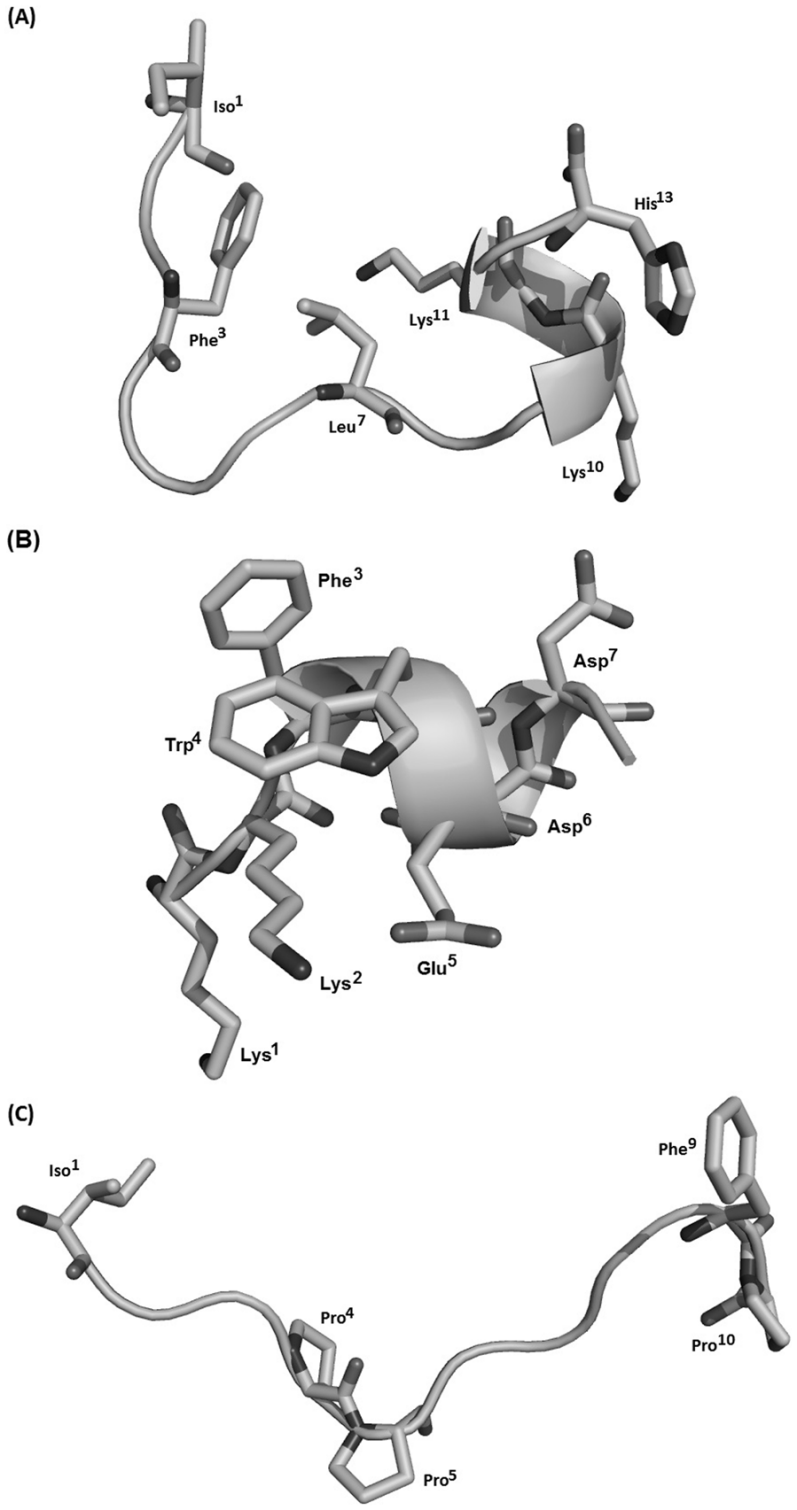
Theoretical three-dimensional structures of peptide derivatives from *Vipera ammodytes ammodytes* L-amino-oxidase (A) BmLAO-f1, (B) BmLAO-f2 and (C) BmLAO-f3. Exposed residues probably involved in membrane interaction are labelled and side chains are also represented.

L-amino acid oxidase (LAO, EC 1.4.3.2) is an enzyme with two subunits. Each subunit consists of 15 α-helices and 22 β-strands that fold into three well-defined domains (Figure 3). These subunits are structurally composed of three domains, a FAD-binding domain, a substrate-binding domain and a helical domain. In this report, the structure of *Vipera ammodytes ammodytes* LAO, previously determined by X-ray crystallography (pdb 3kve) with resolution of 2.57 Å [11], was used as a template for construction of three-dimensional model fragments. All three fragments showed complete identity (100%) with the template. The enzyme also presented theoretical positive charge of +3.5 in accordance with the Protein Calculator v3.3 server (http://www.scripps.edu/~cdputnam/protcalc.html). The fragment model presented random coil conformations (*Bm*LAO-f1 and *Bm*LAO-f3) and also α-helical tendencies (*Bm*LAO-f2) f (Figure 4). The validation of structure fragments was performed by Ramachandran plot, showing that 100% of the amino acid residues from all models are in physically acceptable regions in relation to torsion angles phi and psi. The values of root mean square deviation (RMSD) for peptide fragments were 1.79, 1.64 and 1.80 Å, indicating that the model presented accepted modification and is in accordance with Ramachandran plot. This modification occurs due to side chain liberty in space.

Initially, *Bm*LAO-f1 presented a coil conformation with a hydrophilic and cationic C-terminal composed of Lys^10^, Lys^11^ and His^13^. Furthermore, an α-helical region with net charge +3 was also observed, in accordance with APD prediction describing the structure. The central region is proline-rich with some distributed hydrophobic residues (Phe^3^, Leu^7^) that confer 30% hydrophobic ratio, which probably favours the interaction with phospholipid cell membrane (Figure 4A). In addition, *Bm*LAO-f2 structurally presented a hydrophilic helical conformation composed of Lys^1^, Lys^2^, Glu^5^, Asp^6^ and Asp^7^ that confer a negative charge (−1) and the formation of a dipole helix. One feature that might assist interaction with the membrane is the presence of Phe^3^ and Trp^4^ residues (25% hydrophobic ratio) (Figure 4B). The presence of tryptophan located in the peptide centre might favour a better anchorage by hydrophobic interaction with lipid cell membranes. Finally, *Bm*LAO-f3 structurally presented coil conformation due to the presence of two proline residues (Pro^4^ and Pro^5^) that may make helix formation difficult. The peptide also presents a hydrophobic residue ratio of 22% with an Ile^1^ and Phe^8^ at the N- and C-termini, respectively, which could also contribute to peptide-lipid interaction (Figure 4C).

## Discussion

Currently, HAIs are a major cause of mortality and morbidity among human populations, with wide distribution in many developed and developing countries [1]. Given this fact, fundamental research that aims to develop novel strategies for treating infectious diseases is required, namely using bioactive molecules obtained from natural sources, with direct action on specific cases of these infections. Pitviper venoms contain components with known pharmacological effects, including hypertensive activity, inhibition of platelet aggregation, and anticoagulant, antitumor and antimicrobial activities [3].

In this work, the antimicrobial activity of *B. mattogrosensis* crude venom was evaluated, exhibiting promising antibacterial activity against Gram-positive and -negative bacteria (Table 1). Similar results were presented by San and colleagues [34], observing that venoms from *Calloselasma rhodostoma* and *Ophiophagus hannah* snakes show the ability to inhibit *S. aureus* development. Other crude venom obtained from *Agkistrodon rhodostoma*, *Bothrops jararaca*, *Bothrops atrox*, *Lachesis muta* and *Bothrops alternatus* were also effective against *S. aureus*, *S. epidermidis*, *Enterococcus faecalis* and *E. coli* [35].

In the present work, a LAO was identified as an important component of CVBm antimicrobial activity, also exhibiting antibacterial activity against Gram-positive and -negative bacteria (Table 1). LAO is a multifunctional enzyme that plays an important role in host innate immunity [33], [36], being commonly found in many organisms including bacteria [37], fungi [38], green algae [39], molluscs [40], fish [41], birds [42], reptiles [43] and mammals [44]. In recent years, LAOs with antibacterial activity have been reported from various venoms [6], [7], [45]. LAO enzyme is able to catalyze the oxidation of a large number of amino acids, and this reaction takes place in two key steps. First, the enzyme is reduced by amino acids substrates and then there is a further re-oxidation of the enzyme by molecular oxygen [46]. Data reported indicate that oxidation products of the reaction are an amino acid and also H_2_O_2_. The production of the latter seems to be essentially involved in antibacterial activity. Yang *et al*. [13], working with a LAO from fungal sources (*Trichoderma harzianum*), showed that increased exogenous H_2_O_2_ produced by enzymes and reactive oxidative species accumulated in target bacteria may trigger forms of cell damage, including lipid peroxidation and DNA strand breakage, resulting in bacterial development inhibition. Results indicate that the processes of bacterial interaction, membrane permeabilization and H_2_O_2_ production are directly involved in the mechanism responsible for the antibacterial activity of LAO. Other biological effects have been related to the production of H_2_O_2_ by LAO. The L-amino oxidase synthesized by *Bothrops leucurus* showed the ability to inhibit platelet aggregation as well as exhibiting an *in vitro* lethal effect against Leishmania sp., promastigotes. Moreover, the cytotoxicity of this same compound was observed against stomach cancer MKN-45, adeno carcinoma HUTU, colorectal RKO and human fibroblast LL-24 cell lines [8]. In summary, the enzyme released sufficient H_2_O_2_ in culture medium to induce cellular apoptosis in a dose- and time-dependent manner, corroborating data here reported.

Furthermore, the enzyme sequence here obtained presented 100% of identity with LAO previously isolated from *B. pauloensis* venom [3]. In spite of full identity, it is not possible to conclude for certain that these two enzymes are identical, since a complete sequence was not performed. Moreover, the fragments obtained from *de novo* sequencing were observed in the region that includes 323–420 amino acid residues. In the literature it has been reported that the substrate-binding domain is made up of residues 5–25, 73–129, 233–236 and 323–420 [47]. Recently, Sun and collaborators described a LAO, named Akbu-LAO, which was also characterized by HPLC-ESI-MS/MS showing ten *de novo* sequenced peptides [12]. These peptides presented 100% of identity with the fragments *Bm*LAO-f1 and *Bm*LAO-f2 and showed that the substrate-binding domain is very highly conserved in the LAOs of various snake species. The sequences were in alignment with other LAOs and might indicate that this region is extremely conserved in this protein class.

In recent years, several fragments from larger proteins with antimicrobial properties have been evaluated showing major or similar antimicrobial activities in comparison to complete sequence [48]–[50]. Thennarasu and Nagaraj (1996) [48] examined the antimicrobial and hemolytic activities of the 18-residue segment pardaxin N-terminal. Despite presenting only antimicrobial activity, complete pardaxin shows both antimicrobial and hemolytic activities, indicating that a reductional approach improved bacterial selectivity. Sigurdardottir and colleagues [49] evaluated the activity of three peptides derived from human cathelicidin antimicrobial peptide LL-37. The activities of N- and C-terminal fragments were significant lower than LL-37. However, GKE, a peptide with α-helical propensity, showed higher activity against bacteria and fungi and was less haemolytic than LL-37. More recently, Papareddy *et al.* [50] described several fragments of human thrombin with antimicrobial activity generated by proteolysis. These fragments show similar activities to LL-37, while the complete human thrombin shows no antimicrobial activity.

This work is the first report of fragments of LAO with antimicrobial activity. The fragments here reported showed lower activities in comparison to complete *Bm*LAO. Nonetheless, they could be more useful than *Bm*LAO, since they have a lower amino acid length. Approaches targeting smaller peptides are needed, since they are easier to synthesize, present fewer side effects (such as allergy) and could be less haemolytic [51].

The fragments here reported have unusual properties for antimicrobial peptides in spite of some characteristics that are commonly shared with general AMPs, such as hydrophobicity and amphipathicity. *Bm*LAO-f1 has an unusual proline motif composed of PPLPP between the amino acid 5- and 9-position residues, comparing the *Bm*LAO-f1 to AMPs deposited on APD. This motif is similar to that observed on the PR-bombesin proline-rich peptide, extracted from the toad *Bombina maxima* [52] and which shows *in vitro* activity against Gram-positive, -negative and fungi [53]. Indeed, the sequences are similar, showing 41% of identity. Additionally, *Bm*LAO-f1 shows a net charge of +3 and is composed of 30% of hydrophobic residues, like PR-bombesin, which shows a net charge of +3 and is composed of 31% hydrophobic residues. On the other hand, BmLAO-f1 is not as active as PR-bombesin, probably due to a change in proline motif; PR-bombesin has an arginine residue instead of a leucine in proline motif, which increases the affinity to negatively charged membranes. Besides, an interesting finding is observed when the sequence of PR-bombesin is compared to the mirrored sequence of *Bm*LAO-f1, because the proline motif can be expanded to KKPPXPPX[WF]X[IV], where X can be any of 20 natural amino acids. The lysine residues are probably responsible for bacterial membrane attraction and the proline residues are involved in hydrophobic interactions with the lipid bilayer, as observed for PR-bombesin. Due to proline content, it probably assumes a random coil conformation, like PR-bombesin [53]. Due to the lack of folding, the motif probably works in two orientations, and the great difference between PR-bombesin and BmLAO-f1 is the arginine residue in the middle of the proline residues, present only in PR-bombesin.

*Bm*LAO-f2 is an anionic peptide with charge −1 and moderate ratio hydrophobic content 25%. The peptide presents two lysines (Lys^1^ and Lys^2^) and one tryptophan (Trp^4^). These residues might be important in the interaction with bacterial cell membranes for attraction and anchorage. A similar peptide, named temporin 1Ja, shows a similar net charge (−1) and was isolated from *Rana japonica.* This peptide presented weak activity with MIC values for Gram-negative and - positive bacteria above 100 µM [54], while the peptide produced here was more potent.

Like *Bm*LAO-f1, *Bm*LAO-f3 is also a proline-rich peptide. Nonetheless, *Bm*LAO-f3 is not as hydrophilic and cationic as *Bm*LAO-f1, which probably contributes to its lower activity when compared to *Bm*LAO-f1. *Bm*LAO-f3 has a net charge of +1, reducing its membrane attraction, when compared to *Bm*LAO-f1 (net charge of +3). A peptide's net charge is a well-known property that favours interaction with bacterial membranes through electrostatic interaction [55], [56]. However, its hydrophobic residues compensate for the charge through hydrophobic interactions, mainly the two tyrosines at N-terminal and the phenylalanine at C-terminal. A similar result was reported by Mandal *et al.* [57] in a work which evidenced two peptides (*Cn*AMP-2 and *Cn*AMP-3) with antimicrobial activity from *Cocos nucifera* presenting hydrophobic residues such as tyrosine and phenylalanine [57]. This kind of residue is extremely common in antimicrobial peptides that are well characterized in the literature, such as magainin, fowlicidin, mastoparan and LL-37 [58]–[61]. Furthermore, some peptides showed higher activity toward Gram-negative bacteria than towards Gram-positive ones. In this case, as supported by our data with *Bm*LAO-f1, peptides presented exposed cationic amino acid residues that could play a key role in the interaction with bacterial cell membranes. Several AMPs that target bacteria are cationic and may interact with the anionic lipid components which are exposed on the bacterial membrane. Nevertheless, bacteria diverge extensively in the nature of the major cell membrane lipid components. Those bacteria with anionic and/or zwitterionic or neutral lipids can be induced to form domains in the presence of cationic AMPs [62]. This separation of anionic and zwitterionic lipids into domains can result in bacterial cell death. Such agents are normally more lethal to Gram-negative bacteria, as observed by *Bm*LAO-f1, than to Gram-positive ones, emphasizing the importance of the bacterial membranes' lipid composition in determining susceptibility to antimicrobial agents. Moreover, *in vitro* comparisons between MICs of complete LAO and three fragments suggest that specificity could be related to exposed charges and hydrophobicity. These data demonstrate that other regions with different physicochemical properties could be also attractive for interaction with bacterial cell membranes.

### Conclusions

This is the first report of fragments of the LAO protein from *Bothropoides mattogrosensis* against pathogens such as *B. subtilis*, *E. faecalis*, *S. aureus*, *S. pyogenes*, *E. coli*, *K. pneumonia*, *P. mirabilis*, *P. aeruginosa* and *S. typhimurium*. The activity of these fragments can be improved by modifying the peptide by substitution or deletion of residues. Thus the fragments reported here may be promising candidates in the rational design of new antibiotics that can act against resistant microorganisms.

## Supporting Information

Figure S1 De novo sequencing of peptides generated by mass spectrometer LIFT analysis. (A) BmLAO-f1, (B) BmLAO-f2 and (C) BmLAO-f3 peptides were sequenced through PepSeq.
(TIF)
