# Supplementary figures and images for: Evaluation of an Antimicrobial L-Amino Acid Oxidase and Peptide Derivatives from Bothropoides mattogrosensis Pitviper Venom
Source: PLoS One. 2012 Mar 16;7(3):e33639. doi: 10.1371/journal.pone.0033639 (PMC3306279; doi:10.1371/journal.pone.0033639)

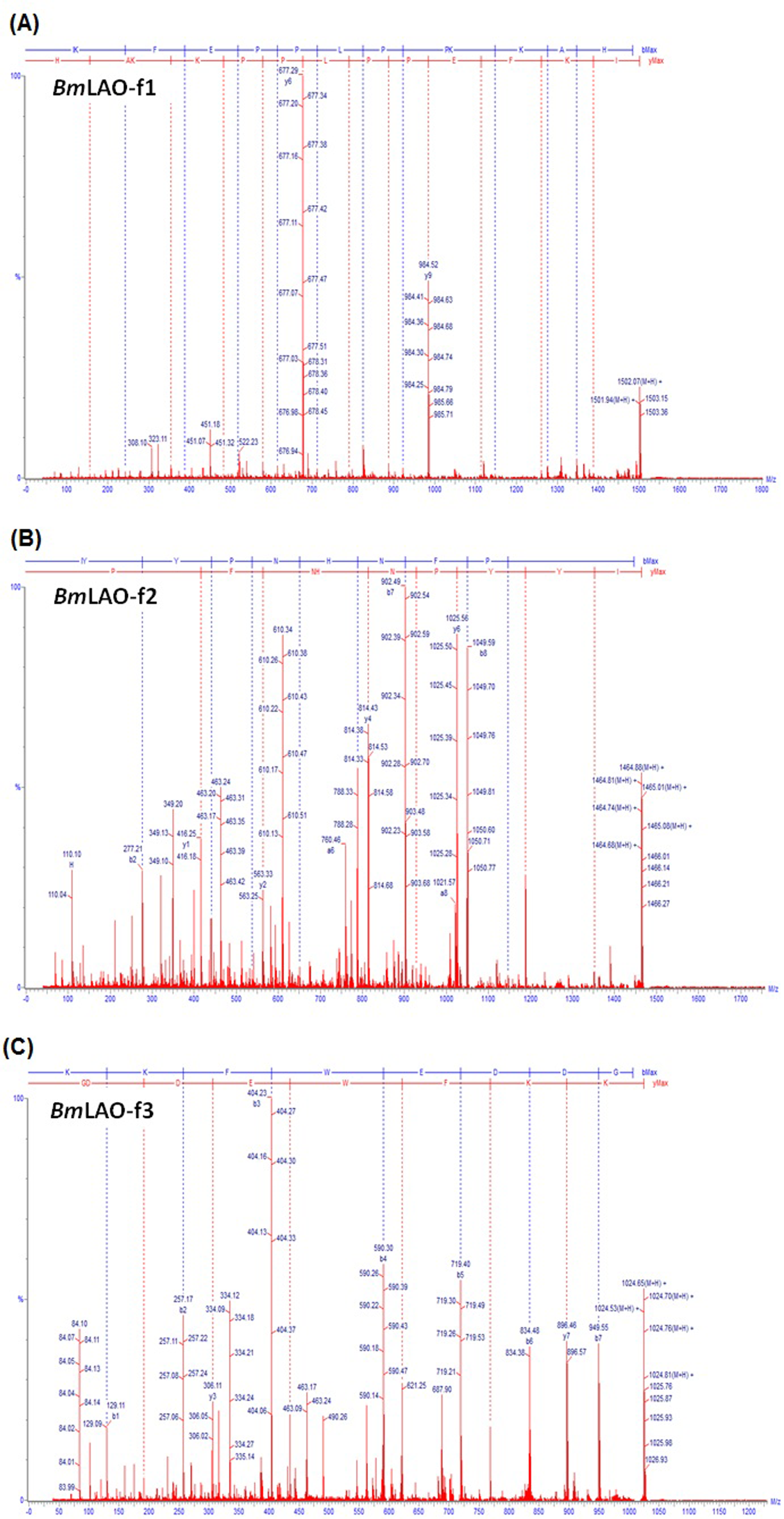

Supplement: Figure S1 — De novo sequencing of peptides generated by mass spectrometer LIFT analysis. (A) BmLAO-f1, (B) BmLAO-f2 and (C) BmLAO-f3 peptides were sequenced through PepSeq. (TIF) [file pone.0033639.s001.tif]
